# Factors Affecting Intraoperative RBC Transfusion in Cerebrovascular Surgery

**DOI:** 10.7759/cureus.99656

**Published:** 2025-12-19

**Authors:** Noriyuki Yahagi, Yushiro Take, Takuma Maeda, Tomomichi Kayahara, Kaima Suzuki, Akaru Ishida, Hiroki Kurita

**Affiliations:** 1 Cerebrovascular Surgery, Saitama Medical University International Medical Center, Hidaka, JPN; 2 Division of Transfusion Medicine and Cell Transplantation, Saitama Medical University International Medical Center, Hidaka, JPN

**Keywords:** anemia, cerebrovascular surgery, preoperative hemoglobin, red blood cell transfusion, red cell concentrate

## Abstract

Purpose

Allogeneic blood transfusion may be required during surgery and can be associated with various adverse effects. However, few studies have investigated the relationship between cerebrovascular surgery and blood transfusions. We aimed to identify factors associated with blood transfusion by comparing the frequency of intraoperative RBC transfusions.

Methods

We retrospectively analyzed the frequency of intraoperative RBC transfusion, excessive RBC transfusion, and intraoperative volume exceeding blood loss in 1,145 patients who underwent cerebrovascular surgery at our institution between January 1, 2019, and November 30, 2023. Patients were divided into elective and emergency cases. Age, sex, surgical procedure, and preoperative hemoglobin (Hgb) levels were used as background factors in the analysis.

Results

In elective surgery, the frequency of intraoperative RBC transfusion was significantly higher in patients undergoing arteriovenous malformation (AVM) removal (P<0.01) and in those with preoperative Hgb levels <12 g/dL (P<0.01). In emergency surgery, intraoperative RBC transfusion was significantly more frequent in patients with preoperative Hgb levels <12 g/dL (P<0.01). The frequency of excessive RBC transfusion during elective surgery was significantly higher in patients with preoperative Hgb levels <12 g/dL (P<0.01). In emergency surgery, excessive RBC transfusion was also significantly more frequent in patients with preoperative Hgb levels <12 g/dL (P<0.01).

Conclusion

Surgical procedure did not affect the frequency of excessive intraoperative RBC transfusion, whereas excessive transfusion was more prevalent in patients with preoperative Hgb levels <12 g/dL. Therefore, preoperative correction of anemia may help reduce this risk.

## Introduction

Patient Blood Management (PBM), introduced around 2010, is an evidence-based approach designed to minimize the use of allogeneic blood transfusions [1]. It is founded on three key pillars: preoperative, intraoperative, and postoperative management [1]. Preoperative management involves the assessment of anemia, iron deficiency, and antithrombotic therapy, with early interventions to correct anemia and optimize hemostasis. Evaluation of cardiopulmonary function is also essential to determine the degree of anemia that can be tolerated. Intraoperative strategies aim to reduce blood loss and transfusion requirements through meticulous surgical techniques, anesthesia methods that minimize bleeding and operative time, and the use of autologous blood salvage devices [2]. Postoperative care focuses on systemic management, blood loss monitoring, oxygen therapy, and appropriate pharmacologic interventions.

Although allogeneic blood transfusions are a limited and valuable resource, they are associated with various adverse effects. The incidence of post-transfusion infections has declined in recent years, and no cases of graft-versus-host disease (GVHD) have been reported since the adoption of irradiation protocols [3]. However, serious complications such as transfusion-related acute lung injury (TRALI) and transfusion-associated circulatory overload (TACO), as well as allergic reactions including urticaria, have been reported [4]. In a large-scale analysis of hospital transfusions, acute transfusion reactions (ATRs) occurred in approximately 0.2% of hospitalizations, with TACO accounting for 0.08% and TRALI for 0.06% of cases [5]. Furthermore, increased transfusion volumes have been linked to a higher risk of systemic infection, postoperative complications, mortality, surgical site infections, and even higher recurrence rates of certain malignancies [6-10].

These risks underscore the importance of minimizing unnecessary transfusions. The concept of restrictive transfusion, which entails administering only the minimum amount of blood required, has been shown to improve patient outcomes, shorten hospital stays, reduce costs, and conserve resources [11, 12]. Despite the extensive literature on blood management in general surgery, few studies have examined transfusion practices specific to neurosurgical procedures, particularly cerebrovascular surgery. Moreover, differences between elective and emergency cases, such as hemodynamic stability, surgical urgency, and preoperative anemia management, may significantly influence transfusion requirements. Therefore, we analyzed these two cohorts separately to clarify their respective determinants. In the present study, we retrospectively analyzed and compared the clinical factors associated with intraoperative RBC transfusions in cerebrovascular surgeries at our institution, with the aim of identifying key determinants of intraoperative transfusion and proposing strategies to reduce unnecessary transfusions in cerebrovascular surgery.

## Materials and methods

We retrospectively analyzed 1,145 cases of cerebrovascular surgery performed at our institution between January 1, 2019, and November 30, 2023, to investigate factors associated with the frequency of intraoperative RBC transfusions. Intraoperative transfusion decisions were made collaboratively by the attending anesthesiologist and neurosurgeon, based on intraoperative blood loss, hemodynamic stability, and laboratory parameters such as hemoglobin (Hgb) or hematocrit levels.

For elective procedures, Fisher’s exact test was used to evaluate the associations between patient characteristics, including age, sex, surgical procedure, and preoperative Hgb level, and the need for intraoperative RBC transfusion. Variables with significant associations in univariate analysis were subsequently entered into a multivariate logistic regression model.

The same analytical approach was applied to emergency procedures, where Fisher’s exact test was performed to evaluate the association between clinical variables and intraoperative RBC transfusion. Emergency procedures were defined as unplanned cerebrovascular operations performed within 24 hours of admission for acute neurological deterioration or life-threatening conditions requiring immediate surgical intervention.

The definition of excessive intraoperative RBC transfusion in this study was based on the ratio of transfusion volume to intraoperative blood loss and did not account for hemodynamic parameters such as blood pressure or heart rate, which could influence transfusion decisions. For elective procedures, factors associated with excessive transfusion were analyzed using Fisher’s exact test and multivariate logistic regression in patients who met this criterion. Similarly, Fisher’s exact test was applied to emergency cases to determine the proportion of patients requiring excessive transfusion and to identify the related factors. All statistical analyses were conducted using EZR (Saitama Medical Center, Jichi Medical University; https://www.jichi.ac.jp/saitama-sct/SaitamaHP.files/statmedEN.html; Kanda, 2012), a graphical user interface for R (The R Foundation for Statistical Computing, Vienna, Austria). Version 1.64 of EZR on R Commander was used. All p-values were two-sided, and p ≤ 0.05 was considered statistically significant. Multicollinearity among independent variables was assessed using the variance inflation factor (VIF), with all variables demonstrating VIF values < 5, indicating no significant multicollinearity.

## Results

Table 1 summarizes the characteristics of 762 elective and 383 emergency cerebrovascular surgeries. The cohort included 88 patients aged 0-40 years, 434 aged 41-65 years, and 623 aged ≥66 years. A total of 491 patients were male and 654 were female. Surgical procedures included 58 cerebral arteriovenous malformation (AVM) removals, 606 aneurysm clippings, 108 carotid endarterectomies (CEA), 130 bypass surgeries, and 243 other procedures (including intracerebral hematoma removal, decompressive craniectomy, cerebral aneurysm wrapping, and hemangioma removal). Preoperative Hgb levels were <12 g/dL in 195 patients and ≥12 g/dL in 950 patients. The intraoperative RBC transfusion rate was 4.1% (31/762) for elective cases and 25.6% (98/383) for emergency cases, with a significantly lower rate in elective procedures.

**Table 1:** Characteristics of all 1,145 cerebrovascular surgery cases at our institution from January 1, 2019, to November 30, 2023. AVM: Arteriovenous malformation; CEA: Carotid endarterectomy; Hgb: Hemoglobin; N: Number of cases; g/dL: Grams per deciliter.

Category		Elective (N = 762)	Emergency (N = 383)
Age (years)	0-40	66 (75%)	22 (25%)
	41-65	292 (67.3%)	142 (32.7%)
	66+	404 (64.8%)	219 (35.2%)
Sex	Male	307 (62.5%)	184 (37.5%)
	Female	455 (69.5%)	199 (30.4%)
Procedure	AVM removal	49 (84.5%)	9 (15.5%)
	Clipping	435 (71.8%)	171 (28.2%)
	CEA	106 (98.1%)	2 (1.9%)
	Bypass	125 (96.2%)	5 (3.8%)
	Others	47 (19.3%)	196 (80.7%)
Hgb level	Hgb < 12.0 g/dL	99 (50.8%)	96 (49.2%)
	Hgb ≥ 12.0 g/dL	663 (69.8%)	287 (30.2%)

Elective surgeries

Table 2 presents the characteristics of the 31 elective patients who required intraoperative RBC transfusion compared with the 731 who did not. Fisher’s exact test demonstrated that transfusion frequency was significantly higher in certain surgical procedure categories (p < 0.01) and in patients with preoperative Hgb <12 g/dL (p < 0.01).

**Table 2:** Comparison of intraoperative RBC transfusion rates in elective operations using Fisher’s exact test. AVM: Arteriovenous malformation; CEA: Carotid endarterectomy; Hgb: Hemoglobin; N: Number of cases; g/dL: Grams per deciliter.

	RBC transfusion, N = 31	No transfusion, N = 731	P-value
Age (years)			
0-40	2 (3.0%)	64 (97.0%)	0.961
41-65	13 (4.5%)	279 (95.5%)	
66+	16 (4.0%)	388 (96.0%)	
Sex			
Male	12 (3.9%)	295 (96.1%)	1
Female	19 (4.2%)	436 (95.8%)	
Procedure			
AVM removal	12 (24.5%)	37 (75.5%)	<0.001
Clipping	12 (2.8%)	423 (97.2%)	
CEA	1 (0.9%)	105 (99.1%)	
Bypass	2 (1.6%)	123 (98.4%)	
Others	4 (8.5%)	43 (91.5%)	
Hgb level			
Hgb < 12.0 g/dL	15 (15.2%)	84 (84.8%)	<0.001
Hgb ≥ 12.0 g/dL	16 (2.4%)	647 (97.6%)	

Multivariate logistic regression analysis identified AVM removal (p < 0.01) and preoperative Hgb <12 g/dL (p < 0.01) as independent predictors of intraoperative RBC transfusion (Table 3). No significant associations were observed for clipping, CEA, bypass, or other procedures.

**Table 3:** Comparison of intraoperative RBC transfusion rates in elective operations using multivariate logistic regression analysis. AVM: Arteriovenous malformation; CEA: Carotid endarterectomy; Hgb: Hemoglobin; g/dL: Grams per deciliter.

Variable	Odds ratios	95% CI	P-value
AVM removal	7.48	1.82-30.8	<0.01
Hgb < 12 g/dL	7.71	3.21-18.5	<0.01
Clipping	0.56	0.15-2.00	0.37
CEA	0.11	0.01-1.08	0.06
Bypass	0.27	0.04-1.68	0.16
Age	1.01	0.98-1.04	0.51
Sex	0.889	0.38-2.09	0.79

Emergency surgeries

Table 4 compares the 98 emergency patients who received intraoperative RBC transfusions with the 285 who did not. The category “others” included procedures such as intracerebral hematoma removal, decompressive craniectomy, cerebral aneurysm wrapping, and hemangioma removal. Fisher’s exact test showed a significantly higher transfusion rate in patients with preoperative Hgb <12 g/dL compared to those with Hgb ≥12 g/dL (p < 0.01). In contrast to elective cases, the type of surgical procedure was not significantly associated with transfusion frequency in emergency cases.

**Table 4:** Comparison of intraoperative RBC transfusion rates in emergency operations using Fisher’s exact test. AVM: Arteriovenous malformation; CEA: Carotid endarterectomy; Hgb: Hemoglobin; N: Number of cases; g/dL: Grams per deciliter.

	RBC transfusion, N = 98	No transfusion, N = 285	P-value
Age (years)			
0-40	5 (22.7%)	17 (77.3%)	0.98
41-65	36 (25.4%)	106 (74.6%)	
66+	57 (26.0%)	162 (74.0%)	
Sex			
Male	43 (23.4%)	141 (76.6%)	0.35
Female	55 (27.6%)	144 (72.4%)	
Procedure			
AVM removal	5 (55.6%)	4 (44.4%)	0.11
Clipping	37 (21.6%)	134 (78.4%)	
CEA	0 (0%)	2 (100%)	
Bypass	2 (40%)	3 (60%)	
Others	54 (27.6%)	142 (72.4%)	
Hgb level			
Hgb < 12.0 g/dL	50 (52.1%)	46 (47.9%)	<0.01
Hgb ≥ 12.0 g/dL	48 (16.7%)	239 (83.3%)	

Excessive transfusion analysis

**Figure 1:**
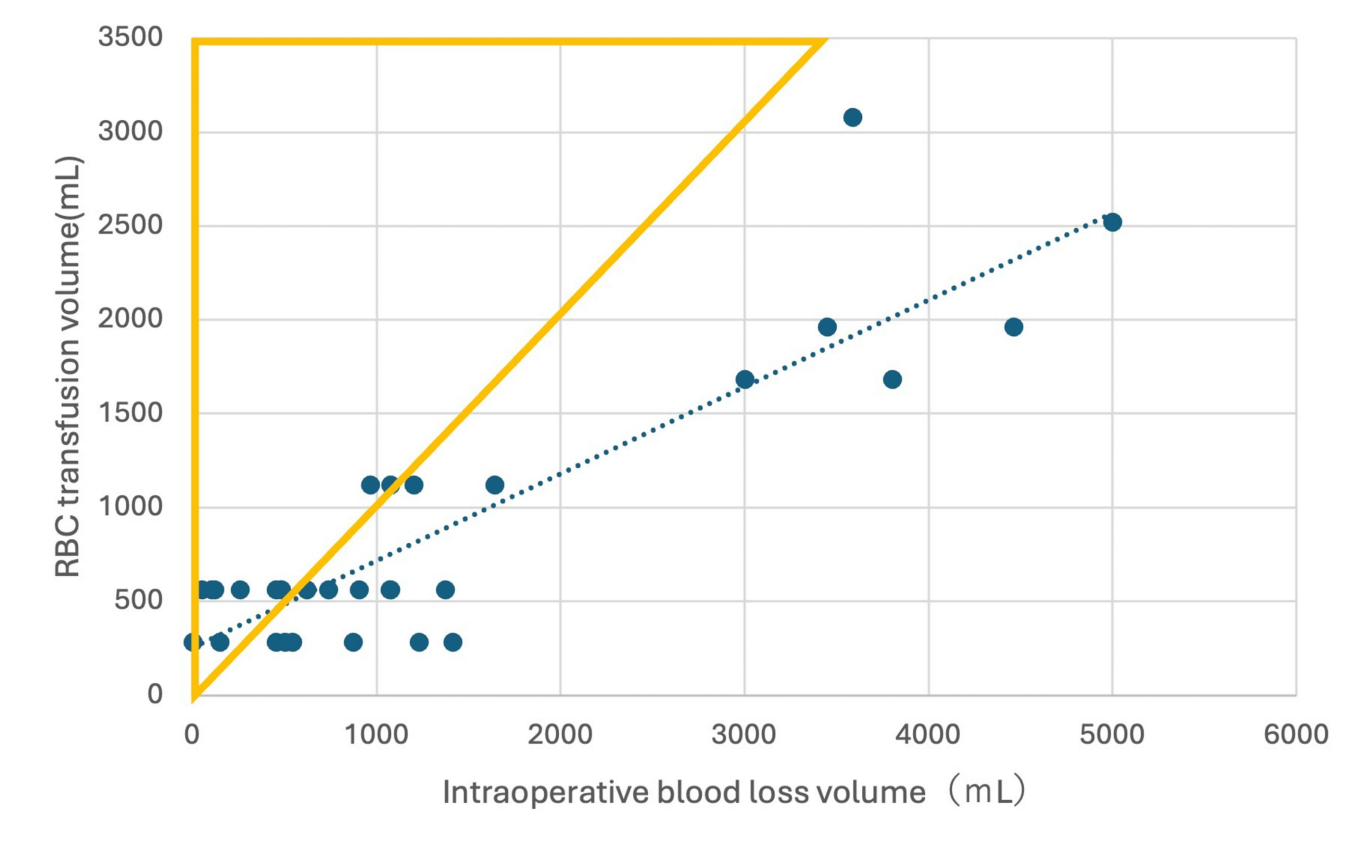
Relationship between intraoperative blood loss and intraoperative RBC transfusion volume in 31 elective cases requiring intraoperative RBC transfusion. Cases with RBC transfusion volume greater than blood loss (n = 11) are shown using yellow triangle markers.

Table 5 details the characteristics of 11 elective cases requiring excessive intraoperative RBC transfusion and 20 cases without excessive transfusion. Excessive transfusion was defined as an intraoperative RBC volume exceeding the measured blood loss. Figure 1 illustrates the relationship between intraoperative blood loss and transfusion volume among the 31 elective patients who received intraoperative RBC transfusions. Univariate analysis revealed a significantly higher frequency of excessive transfusion in patients with preoperative Hgb <12 g/dL (p < 0.01). Surgical procedure type, age, and sex were not significantly associated with excessive transfusion.

**Table 5:** Comparison of intraoperative excessive RBC transfusion rates in elective operations using Fisher’s exact test. AVM: Arteriovenous malformation; CEA: Carotid endarterectomy; Hgb: Hemoglobin; N: Number of cases; g/dL: Grams per deciliter.

	Excessive transfusion, N = 11	Not excessive, N = 20	P-value
Age (years)			
0-40	0 (0%)	2 (100%)	0.27
41-65	3 (23.1%)	10 (76.9%)	
66+	8 (50.0%)	8 (50.0%)	
Sex			
Male	4 (50.0%)	8 (50.0%)	1
Female	7 (36.8%)	12 (63.2%)	
Procedure			
AVM removal	3 (25.0%)	9 (75.0%)	0.57
Clipping	4 (33.3%)	8 (66.7%)	
CEA	1 (100%)	0 (0%)	
Bypass	1 (50.0%)	1 (50.0%)	
Others	2 (50.0%)	2 (50.0%)	
Hgb level			
Hgb < 12.0 g/dL	10 (66.7%)	5 (33.3%)	<0.01
Hgb ≥ 12.0 g/dL	1 (6.2%)	15 (93.8%)	

Multivariate logistic regression analysis confirmed preoperative Hgb <12 g/dL as an independent predictor (p = 0.02) (Table 6). Due to the small number of CEA, bypass, and other cases, these categories were not analyzed as independent variables. Unlike overall transfusion frequency, AVM removal was not significantly associated with excessive transfusion in elective surgeries.

**Table 6:** Comparison of intraoperative excessive RBC transfusion rates in elective operations using multivariate logistic regression analysis. AVM: Arteriovenous malformation; Hgb: Hemoglobin.

Variable	Odds ratios	95% CI	P-value
Hgb < 12.0 g/dL	20	1.62-246.0	0.02
AVM removal	0.59	0.04-9.55	0.71
Clipping	0.62	0.05-7.86	0.71
Sex	0.84	0.10-6.85	0.87
Age	1.03	0.94-1.13	0.53

**Figure 2:**
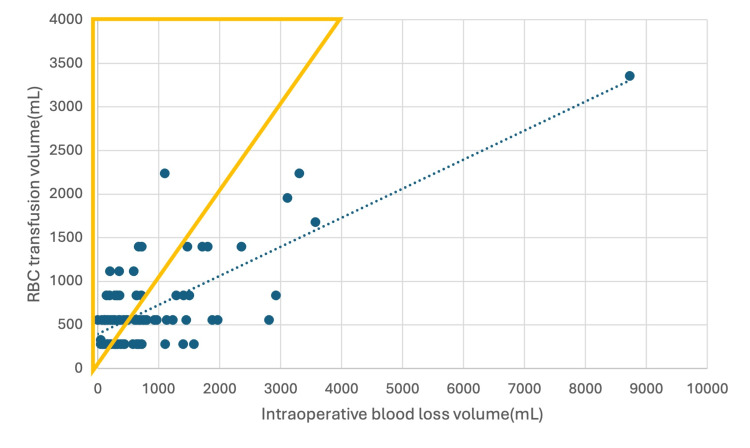
Relationship between intraoperative blood loss and intraoperative RBC transfusion volume in 98 emergency cases requiring intraoperative RBC transfusion. Cases with RBC transfusion volume greater than blood loss (n = 51) are shown using yellow triangle markers.

Table 7 summarizes the characteristics of 51 emergency cases requiring excessive transfusion and 47 without. Figure 2 depicts the relationship between intraoperative blood loss and transfusion volume among the 98 emergency patients who received intraoperative RBC transfusions. In univariate analysis, both age and preoperative Hgb <12 g/dL were significantly associated with excessive intraoperative RBC transfusion in emergency surgeries.

**Table 7:** Comparison of intraoperative excessive RBC transfusion rates in emergency operations using Fisher’s exact test. AVM: Arteriovenous malformation; CEA: Carotid endarterectomy; Hgb: Hemoglobin; N: Number of cases; g/dL: Grams per deciliter.

	Excessive transfusion N = 51	Not excessive N = 47	P-value
Age (years)			
0-40	3 (60.0%)	2 (40.0%)	0.01
41-65	12 (33.3%)	24 (66.7%)	
66+	36 (63.2%)	21 (36.8%)	
Sex			
Male	21 (48.8%)	22 (51.2%)	0.68
Female	30 (54.5%)	25 (45.5%)	
Procedure			
AVM removal	2 (40.0%)	3 (60.0%)	0.87
Clipping	21 (56.8%)	16 (43.2%)	
CEA	0 (0%)	0 (0%)	
Bypass	1 (50.0%)	1 (50.0%)	
Others	27 (50.0%)	27 (50.0%)	
Hgb level			
Hgb < 12.0 g/dL	35 (70.0%)	15 (30.0%)	<0.01
Hgb ≥ 12.0 g/dL	16 (33.3%)	32 (66.7%)	

Across both elective and emergency surgeries, preoperative Hgb <12 g/dL was consistently associated with a higher likelihood of excessive intraoperative RBC transfusion relative to intraoperative blood loss. Age and sex were not significantly associated with excessive transfusion in either group.

## Discussion

In elective cases, factors significantly associated with a higher frequency of intraoperative RBC transfusion were AVM removal and a preoperative Hgb level <12 g/dL. In contrast, in emergency cases, the only significant factor was a preoperative Hgb level <12 g/dL. The absence of a significant association between procedure type and transfusion frequency in emergency cases may reflect the lower likelihood of complex and technically demanding procedures, such as AVM removal, in the emergency setting.

The elevated transfusion frequency observed during AVM removal likely stems from substantial inter-patient variability, as the intraoperative course, from craniotomy to draining vein occlusion and nidus excision, is inherently difficult to predict. AVM removal also induces fluctuations in the hemodynamics of the surrounding brain, which may lead surgeons to adopt a conservative approach to maintaining circulating blood volume. In contrast, for other procedures, experienced neurosurgeons can generally anticipate the operative sequence and expected blood loss, making intraoperative transfusion decisions more dependent on preoperative anemia status when unexpected bleeding does not occur.

In emergency surgery, several factors may contribute to excessive transfusion: preoperative anemia, use of antithrombotic agents, difficulty in accurately estimating intraoperative blood loss due to limited lesion assessment, and the need for frequent irrigation of the surgical field. The latter can result in dilution of blood loss estimates, as cerebrospinal fluid mixes with irrigation solution and blood. Prolonged surgery and inadequate preoperative cardiopulmonary assessment may also increase intraoperative fluid administration, further reducing Hgb concentration.

Procedure type, including AVM removal, was not significantly associated with excessive intraoperative RBC transfusion in either elective or emergency cases. In univariate analysis of elective cases, however, preoperative Hgb <12 g/dL was significantly associated with excessive transfusion; this association persisted in multivariate analysis. These findings support the PBM principle of correcting preoperative anemia. In our cohort, the absence of a significant association between AVM removal and excessive transfusion suggests that transfusion volumes were generally proportional to intraoperative blood loss in these cases.

According to the WHO, anemia is defined as Hgb <13 g/dL in men and <12 g/dL in women. In elective cases, anemia can often be identified and treated in advance through diagnostic work-up and multidisciplinary collaboration. Preoperative anemia has been shown to not only increase intraoperative transfusion requirements but also worsen postoperative outcomes [13]. Proceeding with surgery without correcting anemia may therefore expose patients to unnecessary risks.

The main causes of anemia include iron deficiency, anemia of chronic disease, renal anemia, and deficiencies of folate or vitamin B12. Among these, iron deficiency anemia is the most common and treatable, particularly in older adults [14]. Another third are attributable to anemia of chronic inflammation (ACI) or chronic kidney disease, with the remainder being unexplained. Identifying and correcting reversible causes, through endoscopic evaluation for occult GI bleeding, nutritional assessment, and oral supplementation, should be prioritized. In cases of suspected malignancy-related anemia, hematology consultation is warranted. Preoperative autologous blood donation should be approached cautiously, as anemia induced by pre-donation can negatively affect prognosis, including increased mortality and postoperative complications.

Several randomized controlled trials have demonstrated that restrictive transfusion strategies (Hgb threshold 7-8 g/dL) are non-inferior to liberal strategies (9-12 g/dL) for postoperative outcomes. These findings support the adoption of restrictive thresholds to minimize transfusion-related risks and conserve blood resources without compromising patient safety [11,15,16]. Determining the minimum safe hematocrit and Hgb thresholds preoperatively is essential, yet evidence regarding intraoperative transfusion triggers in neurosurgery, particularly in elderly patients with comorbidities, remains limited. Recent studies have supported the non-inferiority of a restrictive threshold of 8 g/dL in surgical patients [17,18], and some guidelines recommend this approach [19], taking into account surgical stress, coronary artery disease, and physiological reserve. In young, otherwise healthy patients, maintaining circulating blood volume is typically safe with hematocrit levels of 24-27% and Hgb levels of 8.0-9.0 g/dL [20-22]. While physiologically tolerable Hgb levels may be as low as 6.0-7.0 g/dL, patients with reduced cardiopulmonary reserve have less capacity to compensate for blood loss, making preoperative assessment and close anesthesiology collaboration essential.

Limitations

This study has several limitations. First, it was conducted as a retrospective, single-center analysis, which may introduce selection bias and limit generalizability. Second, there was no standardized protocol for intraoperative transfusion within our institution; decisions to transfuse were made at the discretion of the attending anesthesiologists or based on intraoperative requests from neurosurgeons. This operator-dependent variability may have influenced both the frequency and volume of transfusions. Establishing standardized transfusion triggers and criteria would improve the reproducibility of future studies and optimize patient outcomes. Additionally, the definition of excessive intraoperative RBC transfusion in this study was based on a relative comparison between the volume of transfused red blood cells and the measured intraoperative blood loss. Specifically, excessive transfusion was defined as a transfusion volume exceeding the estimated volume of blood lost intraoperatively. This criterion, while pragmatic, may not fully account for individual physiological tolerance, fluid shifts, or surgical context. Therefore, this operational definition may overestimate or underestimate true clinical excess and should be validated in prospective studies using more objective markers, such as post-transfusion Hgb dynamics or hemodynamic parameters.

Intraoperative blood loss was used as a comparative variable to define excessive transfusion and therefore was not included as an independent factor in the statistical models. Future prospective studies should incorporate standardized quantitative blood loss assessment to evaluate its direct influence on transfusion decisions.

Additionally, variables such as intraoperative fluid administration, hemodynamic monitoring data, and transfusion triggers (e.g., Hgb thresholds, cardiac output) were not consistently recorded and could not be analyzed in this dataset. Further multicenter studies with standardized transfusion protocols and broader datasets are warranted.

Finally, the heterogeneity of the procedures categorized as “others” (including intracerebral hematoma removal, decompressive craniectomy, aneurysm wrapping, and hemangioma removal) represents an additional limitation. Because these surgeries vary widely in their complexity, duration, and hemodynamic characteristics, the interpretation of procedure-specific transfusion rates should be approached with caution. Furthermore, in the emergency subgroup, the number of certain procedures, such as AVM removal, CEA, and bypass, was small, which may limit the reliability of statistical comparisons across procedures. Therefore, the interpretation of these results should focus on broader trends, particularly the consistent association between preoperative anemia and transfusion frequency. Overall, these findings should be interpreted as exploratory and hypothesis-generating rather than definitive.

## Conclusions

In elective cerebrovascular surgery, AVM resection and preoperative anemia (Hgb <12 g/dL) independently predicted higher intraoperative RBC transfusion rates. Excessive transfusion, defined as a transfusion volume exceeding intraoperative blood loss, was not procedure dependent but occurred more frequently in patients with preoperative anemia, identifying anemia as a key modifiable driver of transfusion burden. Incorporating routine screening and timely correction of anemia into perioperative pathways may reduce both the frequency and volume of transfusions. Future multicenter studies with standardized, protocol-driven transfusion criteria are warranted to validate these findings and improve reproducibility.
